# Epidemiology of Candidemia in Latin America: A Laboratory-Based Survey

**DOI:** 10.1371/journal.pone.0059373

**Published:** 2013-03-19

**Authors:** Marcio Nucci, Flavio Queiroz-Telles, Tito Alvarado-Matute, Iris Nora Tiraboschi, Jorge Cortes, Jeannete Zurita, Manuel Guzman-Blanco, Maria Elena Santolaya, Luis Thompson, Jose Sifuentes-Osornio, Juan I. Echevarria, Arnaldo L. Colombo, on behalf of the Latin American Invasive Mycosis Network

**Affiliations:** 1 University Hospital, Universidade Federal do Rio de Janeiro, Rio de Janeiro, Brazil; 2 Hospital de Clinicas, Universidade Federal do Parana, Curitiba, Brazil; 3 Hospital Escuela, Tegucigalpa, Honduras; 4 Hospital de Clinicas Jose de San Martin, Buenos Aires, Argentina; 5 Department of Internal Medicine, Universidad Nacional de Colombia, Bogota, Colombia; 6 Hospital Vozandes. Facultad de Medicina, Pontificia Universidad Catolica del Ecuador, Quito, Ecuador; 7 Infectious Diseases, Hospital Vargas de Caracas and Centro Medico de Caracas, Caracas, Venezuela; 8 Department of Pediatrics, Hospital Luis Calvo Mackenna; Faculty of Medicine, Universidad de Chile, Santiago, Chile; 9 Infectious Diseases Unit, Department of Medicine, Clinica Alemana, Universidad del Desarrollo, Santiago, Chile; 10 Instituto Nacional de Ciencias Medicas y Nutrición Salvador Zubiran, Mexico City, Mexico; 11 Department of Medicine, Universidad Peruana Cayetano Heredia, Lima, Peru; 12 Division of Infectious Diseases, Escola Paulista de Medicina, Universidade Federal de São Paulo, São Paulo, Brazil; University of Wisconsin Medical School, United States of America

## Abstract

**Background:**

The epidemiology of candidemia varies depending on the geographic region. Little is known about the epidemiology of candidemia in Latin America.

**Methods:**

We conducted a 24-month laboratory-based survey of candidemia in 20 centers of seven Latin American countries. Incidence rates were calculated and the epidemiology of candidemia was characterized.

**Results:**

Among 672 episodes of candidemia, 297 (44.2%) occurred in children (23.7% younger than 1 year), 36.2% in adults between 19 and 60 years old and 19.6% in elderly patients. The overall incidence was 1.18 cases per 1,000 admissions, and varied across countries, with the highest incidence in Colombia and the lowest in Chile. *Candida albicans* (37.6%), *C. parapsilosis* (26.5%) and *C. tropicalis* (17.6%) were the leading agents, with great variability in species distribution in the different countries. Most isolates were highly susceptible to fluconazole, voriconazole, amphotericin B and anidulafungin. Fluconazole was the most frequent agent used as primary treatment (65.8%), and the overall 30-day survival was 59.3%.

**Conclusions:**

This first large epidemiologic study of candidemia in Latin America showed a high incidence of candidemia, high percentage of children, typical species distribution, with *C. albicans*, *C. parapsilosis* and *C. tropicalis* accounting for the majority of episodes, and low resistance rates.

## Introduction

Candidemia is the leading invasive mycosis occurring in hospitalized patients, with a high burden in tertiary care hospitals worldwide [1], [2]. The epidemiology of candidemia has been extensively described in the Northern Hemisphere, especially in The USA, Western Europe and Asia [3]–[15]. In Latin America, except from Brazil [16]–[21] and other few reports [22]–[24], little is known about the epidemiology of candidemia [25]. The recognition of differences in incidence, populations at greater risk, species distribution and antifungal susceptibility patters is important in order to establish appropriate measures of infection control and for the management of this disease, including prophylaxis and empiric antifungal therapy.

In a nationwide prospective multicenter study conducted in Brazil, a high burden of candidemia was reported, with 2.49 cases per 1,000 admissions (3–10 times higher than that reported in the Northern Hemisphere) with a ∼50% crude mortality rate [19]. In addition, epidemiologic studies in Brazil suggested that *Candida parapsilosis* and *Candida tropicalis* were the most frequent non-*albicans* species, and the proportion of cases due to *Candida glabrata* and *Candida krusei* was low [25].

The Latin America Invasive Mycosis Network is a group of investigators interested in invasive mycoses who met first in 2005 to identify priorities in research and educational activities in the region. In this paper we report the first multicenter prospective epidemiologic study to investigate the epidemiology of candidemia in different Latin American countries.

## Patients and Methods

This is a prospective laboratory-based surveillance study conducted between November 2008 and October 2010 (24 months) in 21 tertiary care hospitals in seven Latin American countries (total 7,445 beds). They were all general hospitals with public (n = 12), private (n = 3) or public and private (n = 6) beds. Sixteen hospitals were for adults and children, one was for adults only and four were for children. All hospitals had intensive care units (ICU, 723 beds), internal medicine and surgery; 18 centers had hematology ward, 10 had solid organ transplantation and 8 had hematopoietic cell transplantation wards. The protocol was approved by ethics committees of each hospital and country without the need of written consent, because of the observational nature of the study: Comite de Etica em Pesquisa da Unifesp/EPM, Comitê de Ética em Pesquisas em Seres Humanos do Hospital de Clínicas da Universidade Federal do Parana, Comite de Etica em Pesquisa do Hospital Pequeno Principe, Comite de Etica em Pesquisa do Hospital Universitário Clementino Fraga Filho (Brazil); Comité de Ética Científico Pediátrico Servicio de Salud Metropolitano Oriente, Comité de Ética Científico Clinica Alemana, Comité de Ética Científico Facultad de Medicina, Universidad de Chile (Chile); Comite de Etica del Hospital de Clinicas Jose de San Martin, Comite de Bioetica, Hospital General de Agudos Juan A. Fernandez, Comite de Etica e Invedstigación, Hospital Pedro de Elizalde (Argentina); Hospital Vozandes Quito Bioethics Committee (Ecuador); Comité de Investigaciones y Ética Institucional (CIEI) de la Facultad De Medicina de la Pontificia Universidad Javeriana y del Hospital Universitario De San Ignacio, Comite de Investigacion, Fundacion Vele del Lili, Comité Independente de Ética en Investigación, Hospital Militar Central, Comité Ético Científico de la Empresa Social del Estado Hospital Universitario de la Samaritana (Colombia), Comite de Etica Centro Medico de Caracas, Comite de Etica Instituto Medico la Floresta (Venezuela), Comite Etica de los Postgrados de Medicina UNAH (Honduras).

Participating institutions carried out laboratory-based sentinel surveillance for candidemia. All hospitals had automated blood culture systems (either Bactec or BacT-ALERT), and an investigator designated to visit the microbiology laboratory on a daily basis in order to capture all episodes of candidemia, and trained to prospectively fill a comprehensive case report form once an episode was diagnosed. The case report form contained detailed information about demographics, underlying conditions, coexisting exposures, receipt of antifungal agents and the outcome. All clinical information was sent using a web-based system (SPSS, Inc.). Audits and query generation were carried out periodically.

All adult and pediatric patients with candidemia were eligible for inclusion in the study, and were followed for 30 days. One episode of candidemia was defined by the isolation of *Candida* species from one or more blood cultures in a patient with clinical signs of infection. If more than one blood culture was positive, a new episode was defined if more than 30 days had elapsed since the first positive blood culture (incident candidemia). Breakthrough candidemia was defined when the patient was receiving a systemic antifungal agent for >3 days once candidemia was diagnosed. Neonates were defined as patients with age ≤28 days, infants as children aged >28 days to 1 year, older children as patients 1–18 years old, adults as patients 19–60 years old, and elderly as patients >60 years.

All isolates were identified at species level in the local laboratory, but were also sent to the Special Mycology Laboratory (Universidade Federal de São Paulo) for confirmation of species, as well as antifungal susceptibility tests. Isolates were identified according to their microscopic morphology on cornmeal Tween 80 agar, complemented by biochemical tests using the ID 32C system (BioMérieux AS, Marcy ĺ Etoile, France). Sequencing of the ITS region of ribosomal DNA was used to identify species other than *C. albicans*, *C. parapsilosis*, *C. tropicalis*, *C. glabrata* and *C. krusei*. Antifungal susceptibility tests were performed using a broth microdilution assay following the methods recommended by the Clinical and Laboratory Standards Institute (CLSI) [26]. The following antifungal drugs were tested: fluconazole (Pfizer Incorporated, New York, NY, USA), amphotericin B (Sigma Chemical Corporation, St Louis, MO, USA), voriconazole and anidulafungin (Pfizer Incorporated, New York, NY, USA). The assays were incubated at 35°C for 24 h. The minimum inhibitory concentration (MIC) breakpoints for fluconazole were: for *Candida albicans*, *C. parapsilosis* and *C. tropicalis* isolates with MIC ≤2 µg/ml were considered susceptible, those with MIC 4 µg/ml were considered susceptible dose-dependent (SDD), and those with MIC ≥8 µg/ml were considered resistant; for *C. glabrata* isolates with MIC ≤32 µg/ml were considered SDD, and MIC ≥64 µg/ml were considered resistant. All *C. krusei* isolates were considered resistant regardless of the MIC value. For voriconazole, isolates with MIC ≤2 µg/ml were considered susceptible, those with MIC 4 µg/ml were considered susceptible dose-dependent (SDD), and those with MIC ≥8 µg/ml were considered resistant. For anidulafungin, isolates of *C. albicans*, *C. tropicalis* and *C. krusei* with MIC ≤0.25 µg/ml were considered susceptible, those with MIC 0.5 µg/ml were intermediate, and isolates with MIC ≥1 µg/ml were considered resistant. For *C. parapsilosis* isolates with MIC ≤2 µg/ml were susceptible, 4 µg/ml were intermediate, and ≥8 µg/ml were resistant. For *C. glabrata* the values were as follows: ≤0.12 µg/ml susceptible, 0.25 µg/ml intermediate, and ≥0.5 µg/ml resistant. For amphotericin B, MICs ≤1 µg/ml were considered susceptible and those ≥2 µg/ml were considered resistant [27].

Incidence density of candidemia was calculated using the number of episodes of candidemia as numerators, and admissions and patients-day as denominators. Incidence rates were calculated for each hospital, each country and overall. Dichotomous variables were compared using Fisher or Chi-square test, as appropriate, and continuous variables were compared using the Wilcoxon test. All statistical analyses were performed in the SPSS software (version 15, SPSS, Inc.). P values <0.05 were considered statistically significant.

## Results

During the study period 672 episodes of candidemia were reported. The median age was 26 years (0–98), and 58.9% were males. Two hundred and ninety seven episodes (44.2%) occurred in children (23.7% younger than 1 year, including 89 neonates, and 20.5% between 1 and 18 years), 36.2% in adults between 19 and 60 years old and 19.6% in elderly patients. Excluding the four children hospitals the proportion of children in the cohort was still high (31.2%). The overall incidence was 1.18 cases per 1,000 admissions and 0.23 cases per 1,000 patients-day. As shown in Table 1, the incidence differed across countries, with the highest incidence in Argentina (1.95 cases per 1,000 admissions and 0.24 cases per 1,000 patients-day) and the lowest in Chile (0.33 per 1,000 admissions and 0.09 per 1,000 patients-day). There was also a great variability in the incidence among the centers; the highest incidence was in an Argentinian hospital with 2.98 cases per 1,000 admissions, and the lowest in a Chilean center (0.21 per 1,000 admissions).

**Table 1:** Incidence of candidemia in 20 hospitals of 7 Latin American Countries.

Country	Incidence per 1,000 admissions (range)	Incidence per 1,000 patients.day (range)
Argentina	1.95 (1.26–2.98)	0.24 (0.13–0.39)
Brazil	1.38 (0.55–2.11)	0.26 (0.14–0.30)
Chile	0.33 (0.21–0.47)	0.09 (0.06–0.16)
Colombia	1.96*	0.16 (0.12–0.24)
Ecuador	0.90 (0.30–1.10)	0.16 (0.10–0.17)
Honduras	0.90 (0.88–0.98)	0.25 (0.24–0.30)
Venezuela	1.72 (1.04–2.90)	NA
Overall	1.18	0.23

NA = not available.

* information on number of admission was available in only one hospital.

As shown in Table 2, *C. albicans* was the leading agent (37.6%), followed by *C. parapsilosis* (26.5%), *C. tropicalis* (17.6%), *C. guilliermondii* (6.5%), *C. glabrata* (6.3%), and *C. krusei* (2.7%). There was a great variability in species distribution in the different countries. Ecuador had the highest proportion of episodes due to *C. albicans* (52.2%) and Honduras and Venezuela the lowest (27.4% and 26.8%, respectively). While *C. parapsilosis* was highly prevalent in most countries, it was less frequent in Honduras (14.1%). By contrast, *C. guilliermondii* was very common in this country (20.7%). The highest proportion of episodes caused by *C. glabrata* was observed in Brazil (10%) and the lowest was seen in Venezuela (1 episode, 2.4%).

**Table 2:** Species distribution of 672 episodes of candidemia.

	Argentina	Brazil	Chile	Colombia	Ecuador	Honduras	Venezuela	Overall
*C. albicans*	48 (42.5)	77 (40.5)	16 (42.1)	40 (36.7)	24 (52.2)	37 (27.4)	11 (26.8)	253 (37.6)
*C. parapsilosis*	27 (23.9)	49 (25.8)	11 (28.9)	42 (38.5)	14 (30.4)	19 (14.1)	16 (39.0)	178 (26.5)
*C. tropicalis*	19 (16.8)	25 (13.2)	4 (10.5)	19 (17.4)	5 (10.9)	36 (26.7)	10 (24.4)	118 (17.6)
*C. guilliermondii*	7 (6.2)	3 (1.6)	1 (2.6)	2 (1.8)	1 (2.2)	28 (20.7)	2 (4.9)	44 (6.5)
*C. glabrata*	7 (6.2)	19 (10.0)	3 (7.9)	5 (4.6)	2 (4.3)	5 (3.7)	1 (2.4)	42 (6.3)
*C. krusei*	2 (1.8)	9 (4.7)	3 (7.9)	–	–	4 (3.0)	–	18 (2.7)
Other*	3	8	–	1	–	6	1	19 (2.8)
Total	113	190	38	109	46	135	41	672

* Other species – Argentina: *C. lusitaniae* (2), *C. pelliculosa* (1); Brazil: *C. intermedia* (3), *C. haemulonii* (2), *C. lusitaniae*, *C. famata*, *C. norvegiensis* (1 each); Colombia: *C. lusitaniae* (1); Honduras: *C. lusitaniae* (3), *C. pelliculosa*, *C. haemulonii*, *C. albicans+C. glabrata* (1 each); Venezuela: *C. pelliculosa* (1).

Overall, cancer was the most frequent underlying condition (22.5%), and 43.8% of episodes occurred in the context of recent surgery (within 3 months of the incident candidemia). The median APACHE II score was 18 (data available in 258 episodes) and 44.6% were in an ICU at the time of the incident candidemia. The median duration of hospitalization before candidemia was 15 days (0–176).

As shown in Table 3, characteristics of the episodes of candidemia differed according to the age strata. For example, ICU admission was more frequent in neonates, elderly patients and infants, and less frequent in children (p<0.001); hematologic malignancies were more prevalent in children while solid tumors were more frequent in elderly (p<0.001). Co-morbidities like cardiac, neurologic, lung, liver disease, chronic renal failure and diabetes were more frequent in elderly patients. By contrast, the proportion of patients undergoing abdominal surgery was very similar across age strata.

**Table 3:** Characteristics of 672 episodes of candidemia by age strata.

	NeonatesN = 89	InfantsN = 70	Older childrenN = 138	AdultsN = 223	ElderlyN = 152	OverallN = 672
Gender, male : female	41∶48	54∶16	82∶56	132∶91	87∶65	396∶276
Age*, median (range)	16 (1–28)	4 (1–11)	5 (1–18)	45 (19–60)	76 (61–98)	26 (0–98)
APACHE II score**, median (range)	15 (6–24)	13.5 (8–24)	18 (4–21)	16 (2–38)	21.5 (10–39)	18 (2–39)
Duration (days) of hospitalization before candidemia, median (range)	12 (0–28)	19 (0–146)	13 (0–176)	17 (0–162)	20 (0–130)	15 (0–176)
Admission to an ICU	70 (78.7)	34 (48.6)	36 (26.1)	83 (36.8)	78 (51.3)	300 (44.6)
Cancer	–	4 (5.7)	46 (33.3)	60 (26.9)	41 (27.0)	151 (22.5)
Hematological malignancy	–	1 (1.4)	31 (22.5)	29 (13.0)	8 (5.3)	69 (10.3)
Solid tumor	–	3 (4.3)	15 (10.9)	31 (13.9)	33 (21.7)	82 (12.2)
Cardiac disease	18 (20.2)	15 (21.4)	14 (10.1)	34 (15.2)	76 (50.0)	157 (23.4)
Neurological disease	6 (6.7)	11 (5.7)	27 (19.6)	31 (13.9)	43 (28.3)	118 (17.6)
Lung disease	27 (30.3)	19 (27.1)	15 (10.9)	46 (20.6)	60 (39.5)	167 (24.9)
Diabetes mellitus	1 (1.1)	–	4 (2.9)	33 (14.8)	38 (25.0)	76 (11.3)
Renal failure	10 (11.2)	10 (14.3)	19 (13.8)	55 (24.7)	56 (36.8)	150 (22.3)
Chronic renal failure	–	1 (1.4)	9 (6.5)	24 (10.8)	24 (15.8)	58 (8.6)
Liver disease	2 (2.2)	6 (8.6)	8 (5.8)	34 (15.2)	18 (11.8)	68 (10.1)
Surgery	26 (29.2)	27 (38.6)	58 (42.0)	105 (47.1)	78 (51.3)	294 (43.8)
Abdominal surgery	18 (20.2)	17 (24.3)	29 (21.0)	54 (24.2)	38 (25.0)	156 (23.2)
Mechanical ventilation	60 (67.4)	31 (44.3)	32 (23.2)	102 (45.7)	82 (53.9)	307 (45.7)
Parenteral nutrition	43 (48.3)	18 (25.7)	26 (18.8)	64 (28.7)	41 (27.0)	192 (28.6)
Dialysis	3 (3.4)	3 (4.3)	8 (5.8)	35 (15.7)	27 (17.8)	76 (11.3)
Neutropenia	1 (1.1)	4 (5.7)	30 (21.7)	19 (8.5)	3 (2.0)	57 (8.5)
Central venous catheter	63 (70.8)	38 (54.3)	91 (65.9)	167 (74.9)	124 (81.6)	483 (71.9)
Receipt of antibiotic	85 (95.5)	68 (97.1)	131 (94.9)	211 (94.6)	140 (92.1)	635 (94.5)
Receipt of corticosteroids	12 (13.5)	16 (22.9)	48 (34.8)	87 (39.0)	62 (40.8)	225 (33.5)
Previous use of fluconazole	15 (16.9)	3 (4.3)	22 (15.9)	48 (21.5)	19 (12.5)	107 (15.9)

NOTE: Numbers are no. (%) of patients, unless otherwise indicated. APACHE = Acute Physiologic and Chronic Health Evaluation;

Infants defined as children older than 28 days and younger than 1 year; Elderly defined as patients older than 60 years.

* days for neonates, months for infants and years for the other age strata and overall.

** data available for 258 episodes.

Species distribution was also different (Table 4). For example, the proportion of candidemia due to *C. glabrata* was significantly higher in elderly patients (15.1% vs. 3.4% in neonates, 1.4% in infants, 3.6% in children and 4.5% in adults, p<0.001). No other variable besides age was associated with *C. glabrata* candidemia, including prior fluconazole use (3.5% with vs. 6.6% without prior fluconazole exposure, p = 0.20). For *C. guilliermondii*, the rates in children were significantly higher (11.6%) than in other age strata, whereas *C. tropicalis* was more frequent in adults. By contrast, there was no association between age and *C. parapsilosis* candidemia, with high rates in all age strata.

**Table 4:** Species distribution and treatment of 672 episodes of candidemia by age strata.

	NeonatesN = 89	InfantsN = 70	Older childrenN = 138	AdultsN = 223	ElderlyN = 152	OverallN = 672
Species distribution						
* Candida albicans*	39 (43.8)	34 (48.6)	41 (29.7)	75 (33.6)	64 (42.1)	253 (37.6)
* Candida parapsilosis*	24 (27.0)	17 (24.3)	39 (28.3)	67 (30.0)	31 (20.4)	178 (26.5)
* Candida tropicalis*	13 (14.6)	6 (8.6)	22 (15.9)	49 (22.0)	28 (18.4)	118 (17.6)
* Candida guilliermondii*	4 (4.5)	11 (15.7)	16 (11.6)	9 (4.0)	4 (2.6)	44 (6.5)
* Candida glabrata*	3 (3.4)	1 (1.4)	5 (3.6)	10 (4.5)	23 (15.1)	42 (6.3)
* Candida krusei*	4 (4.5)	–	5 (3.6)	9 (4.0)	–	18 (2.7)
Received antifungal therapy	71 (79.8)	64 (91.4)	122 (88.4)	191 (85.6)	126 (82.9)	574 (85.4)
Primary treatment*						
Fluconazole	25/71 (35.2)	37/64 (57.8)	76/122 (62.3)	141/191 (73.8)	99/126 (78.6)	378/574 (65.8)
d-AMB	39/71 (54.9)	21/64 (32.8)	39/122 (32.0)	27/191 (14.1)	13/126 (10.3)	139/574 (24.2)
Caspofungin	4/71 (5.6)	–	3/122 (2.4)	14/191 (7.3)	8/126 (6.3)	29/574 (5.0)
Anidulafungin	–	3/64 (4.7)	1/122 (0.8)	3/191 (1.6)	4/126 (3.2)	11/574 (1.9)
Voriconazole	2/71 (2.8)	1/64 (1.6)	1/122 (0.8)	6/191 (3.1)	2/126 (1.6)	12/574 (2.1)
l-AMB	1/71 (1.4)	2/64 (3.1)	2/122 (1.6)	–	–	5/574 (0.9)
15-day survival**	56 (64.4)	47 (74.6)	95 (76.0)	137 (64.9)	56 (39.4)	391 (62.3)
30-day survival***	46 (59.7)	44 (73.3)	84 (73.7)	120 (61.9)	52 (37.7)	346 (59.3)

NOTE: Numbers are no. (%) of patients; d-AMB = deoxycholate amphotericin B; l-AMB = lipid formulation of amphotericin B.

Infants defined as children older than 28 days and younger than 1 year; Elderly defined as patients older than 60 years.

* In 12 episodes no information was available regarding antifungal treatment.

** Data available in 628 episodes.

*** Data available in 583 episodes.

Information on antifungal treatment was available in all but 12 episodes (Table 4). Antifungal treatment was given in 85.4% of episodes, at a median of two days from the date of the incident candidemia (0–26). In 61 of the 86 (70.9%) episodes of candidemia in which no antifungal treatment was given, death occurred within five days from the date of the incident candidemia. Fluconazole was the most frequent agent used as primary treatment (65.8%), followed by deoxycholate amphotericin B (24.2%) and an echinocandin (6.9%). There was an inverse relationship between age strata and receipt of deoxycholate amphotericin B as primary therapy for candidemia: 54.9% in neonates, 32.8% in infants, 32% in older children, 14.1% in adults and 10.3% only in elderly patients (p<0.001).

The 30-day survival (data available in 583 episodes) was 59.3% (Table 4). Elderly patients had the lowest 30-day survival rate (37.7%), followed by neonates (59.7%), adults (61.9%), infants (73.3%) and older children (73.7%). The 30-day survival among treated patients was 65.3%, and was not different according to the antifungal agent used as primary treatment.

Table 5 summarizes the antifungal susceptibility tests of the most frequent *Candida* bloodstream isolates. For fluconazole, resistance was observed only in *C. krusei* (all resistant by definition) and *C. glabrata* (7.1%). In addition, SDD was observed in one *C. albicans* (0.4%), two *C. parapsilosis* (1.1%), and 39 (92.9%) *C. glabrata* isolates. All isolates were susceptible to voriconazole and amphotericin B. For anidulafungin, there were two (4.8%) *C. glabrata* isolates, one (0.4%) *C. albicans* and two (1.7%) *C. tropicalis* isolates with intermediate susceptibility.

**Table 5:** *In vitro* susceptibility of *Candida* species to four antifungal agents.

Species (N)	Antifungal agent	MIC* (µg/ml)			SDD or I, n (%)	Resistant, n (%)
		Range	MIC 50	MIC 90		
*C. albicans* (253)	Amphotericin B	0.125–1.0	0.5	1.0	NA	0
	Fluconazole	0.125–4.0	0.125	0.5	1 (0.4)	0
	Voriconazole	0.03–0.125	0.03	0.03	0	0
	Anidulafungin	0.03–0.5	0.03	0.125	1 (0.4)	0
*C. parapsilosis* (178)	Amphotericin B	0.25–1.0	0.5	1.0	NA	0
	Fluconazole	0.125–4.0	0.25	1.0	2 (1.1)	0
	Voriconazole	0.03–0.25	0.03	0.25	0	0
	Anidulafungin	0.03–2.0	1.0	2.0	0	0
*C. tropicalis* (118)	Amphotericin B	0.25–1.0	0.5	1.0	NA	0
	Fluconazole	0.125–1.0	0.25	0.5	0	0
	Voriconazole	0.03–0.125	0.03	0.03	0	0
	Anidulafungin	0.03–0.5	0.03	0.25	2 (1.7)	0
*C. guilliermondii* (44)	Amphotericin B	0.25–1,0	0.5	1.0	NA	0
	Fluconazole	0.125–16	2.0	8.0	ND	ND
	Voriconazole	0.03–0.5	0.06	0.25	0	0
	Anidulafungin	0.03–2.0	1.0	2.0	ND	ND
*C. glabrata* (42)	Amphotericin B	0.25–1.0	0.5	1.0	NA	0
	Fluconazole	0.25–64	4.0	16	39 (92.9)	3 (7.1)
	Voriconazole	0.03–2.0	0.125	0.5	0	0
	Anidulafungin	0.03–0.25	0.06	0.125	2 (4.8)	0
*C. krusei* (18)	Amphotericin B	0.5–1.0	0.5	1.0	NA	0
	Fluconazole	8.0–32	16	32	0	18 (100)
	Voriconazole	0.03–0.25	0.125	0.25	0	0
	Anidulafungin	0.03–0.125	0.06	0.125	0	0

MIC = minimal inhibitory concentration; SDD = susceptible, dose-dependent; I = intermediate; NA = not applicable; ND = not defined.

## Discussion

In this first multi-country epidemiologic study of candidemia in Latin America we confirmed some unique epidemiologic features reported in multicenter studies in Brazil: high incidence, a large proportion of children (not only neonates), the typical species distribution with high rates of *C. tropicalis* and *C. parapsilosis* and low rate of *C. glabrata* candidemia, and low resistance rates. In addition, we observed some important differences in the epidemiology across countries.

The proportion of children in this cohort was very high (44.2%). This is in sharp contrast with series from Europe and the USA. For example, a 28-month prospective study in six European countries reported 7.6% of children in a cohort of 2,089 episodes of candidemia [28]. Other European series reported rates between 2 and 9% [11], [12], [29], [30], and a large prospective study in the USA reported 9% of children among 1,591 episodes of candidemia [14]. Another important difference is that while in those series the majority of children were neonates, in the present series there was a high proportion of infants (23.6% of the pediatric population).

We observed a high burden of candidemia in the region, with 1.19 cases per 1,000 admissions. The incidence rates were higher than those reported in the Northern Hemisphere [11], [13], [15], [28], [31]–[33], but lower compared to Brazilian studies. A multicenter study reported 2.49 cases per 1,000 admissions [19], but the rates were even higher in more recent single-center studies (3.6 to 6.0 in one study [34] and 1.54 to 2.99 in another [35]). However, lower incidence rates (0.74–0.91 cases per 1,000 admissions) were observed in private hospitals in this country [36], [37]. In the present study, three hospitals were private and six had both public and private coverage. Therefore, it is possible that these differences may partly explain the great variety in the incidence of candidemia observed across countries and across centers within a country. Although speculative, other factors that may have contributed to this variation include the characteristics of the hospitals, with different patient populations and standards of prophylaxis and empiric therapy.

In our study, species distribution confirmed that in Latin America, *C. albicans*, *C. parapsilosis* and *C. tropicalis* account for more than 80% of episodes of candidemia, *C. parapsilosis* is not clustered in children, and the frequency of *C. glabrata* candidemia is lower than that reported in the Northern Hemisphere [25]. However, there were some important differences across countries. For example, Brazil had the highest proportion of candidemia due to *C. glabrata* (10%). A trend for an increase in the incidence of *C. glabrata* candidemia in Brazilian hospitals has been recently reported in two studies. In a single-center study, the proportion of *C. glabrata* increased from 4.8% in 2008 to 23.5% in 2010. No association between fluconazole use and the increase in *C. glabrata* candidemia was observed [34]. By contrast, another study observed a higher proportion of candidemia due to *C. glabrata* in private hospitals compared to public hospitals. Patients from private hospitals were more likely to have been exposed to fluconazole, and *C. glabrata* isolates from private hospitals were less susceptible to fluconazole [37]. In the present study, we did not observe an association between fluconazole exposure and candidemia due to *C. glabrata*. On the other hand, *C. glabrata* candidemia was more frequent in elderly patients, as reported elsewhere [38]. Interestingly, Brazilian patients (the country with the highest proportion of *C. glabrata*) were significantly older than patients from other countries (median age 45 years vs. 20 years, p<0.001). Furthermore, the proportion of isolates with resistance to fluconazole was low (6.5% only). Taken together, it seems that there are two epidemiologic scenarios of candidemia due to *C. glabrata*: one that is driven by older age, typically with more fluconazole-susceptible isolates, and other driven by selective pressure of fluconazole use, with less-susceptible isolates. More studies are needed to confirm this hypothesis.

Our study confirmed that *C. parapsilosis* is very prevalent in Latin America, and is distributed in all age strata, contrasting to studies from other parts of the globe, in which this species is more frequent among neonates [39]. We also observed a high proportion of candidemia episodes caused by *C. guilliermondii*. The majority of cases occurred in Honduras, mostly in children. A pseudo-outbreak of *C. guilliermondii* candidemia was reported in a center in Brazil, with most cases occurring in pediatric patients [40]. The pseudo-outbreak was suspected because of the cluster of cases in time and place, and also because the large majority of patients did not have the typical risk factors for candidemia or had clinical manifestations of infection. In the present study, we performed a careful review of the clinical characteristics of the cases, including risk factors, antifungal treatment and outcome, and did not find any feature suggestive of a pseudo-outbreak.

The antifungal susceptibility tests confirmed previous studies [25], with very low rates of resistance, except for *C. glabrata* and *C. krusei* and fluconazole. In addition, we observed two *C. glabrata* isolates (4.3%) exhibiting higher MICs to anidulafungin. The clinical relevance of these findings is unknown because although epidemiologic breakpoints for the echinocandins have been proposed [26], the correlation between MIC and the outcome is still uncertain. However, caution should be taken since sporadic cases of therapeutic failure with echinocandins have been reported in candidemia due to *C. glabrata* [41], and data on an animal model suggested that the echinocandins have a fungistatic effect against this species [42].

In the present study, almost 15% of patients did not receive treatment. The most likely reason was late diagnosis, since the majority of such patients died very early after the incident candidemia. Fluconazole was the leading agent given as primary treatment. Echinocandins were used infrequently despite their excellent attributes for the treatment of candidemia, probably because of its high price compared with deoxycholate amphotericin B and fluconazole. Another interesting observation was that elderly patients were much less likely to have received deoxycholate amphotericin B as primary therapy.

A limitation of our study is that although 21 centers from seven Latin American countries participated, this may not be representative of each country, especially those with higher population and bigger territory, such as Argentina and Brazil. Nevertheless, this is the first attempt to estimate the burden and to characterize the epidemiology of candidemia in the region. Additional studies are needed to expand the epidemiology of candidemia in individual countries, especially those with no data. In addition, a better characterization of the epidemiology of candidemia in the pediatric population is warranted, considering the high burden and consequences of candidemia in these patients.

In conclusion, this first large epidemiologic study of candidemia in Latin America showed similar findings as studies conducted in Brazil: high incidence, high percentage of children, typical species distribution, with *C. albicans*, *C. parapsilosis* and *C. tropicalis* accounting for the majority of episodes, and low resistance rates.
